# Supplementary material for: Dirac cone, flat band and saddle point in kagome magnet YMn6Sn6
Source: Nat Commun. 2021 May 25;12:3129. doi: 10.1038/s41467-021-23536-8 (PMC8149840; doi:10.1038/s41467-021-23536-8)
Supplement: Supplementary file 1 — Supplementary Information [file 41467_2021_23536_MOESM1_ESM.pdf]

Supplementary Information:

Dirac cone, flat band and saddle point in kagome magnet YMn_6Sn_6

Li et al.

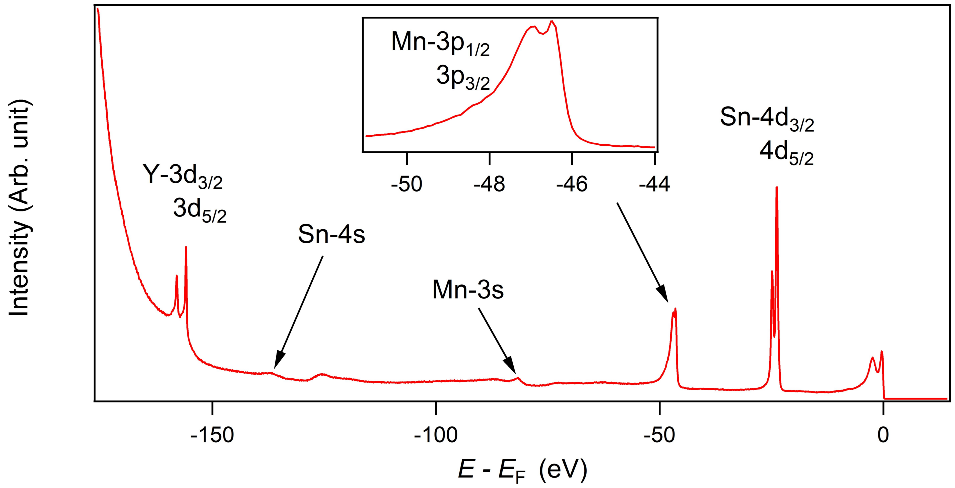

Supplementary Figure 1. Core-level spectrum of YMn_6Sn_6 taken with $h\nu$ = 200 eV. The total angular momentum quantum numbers are labelled for peaks.

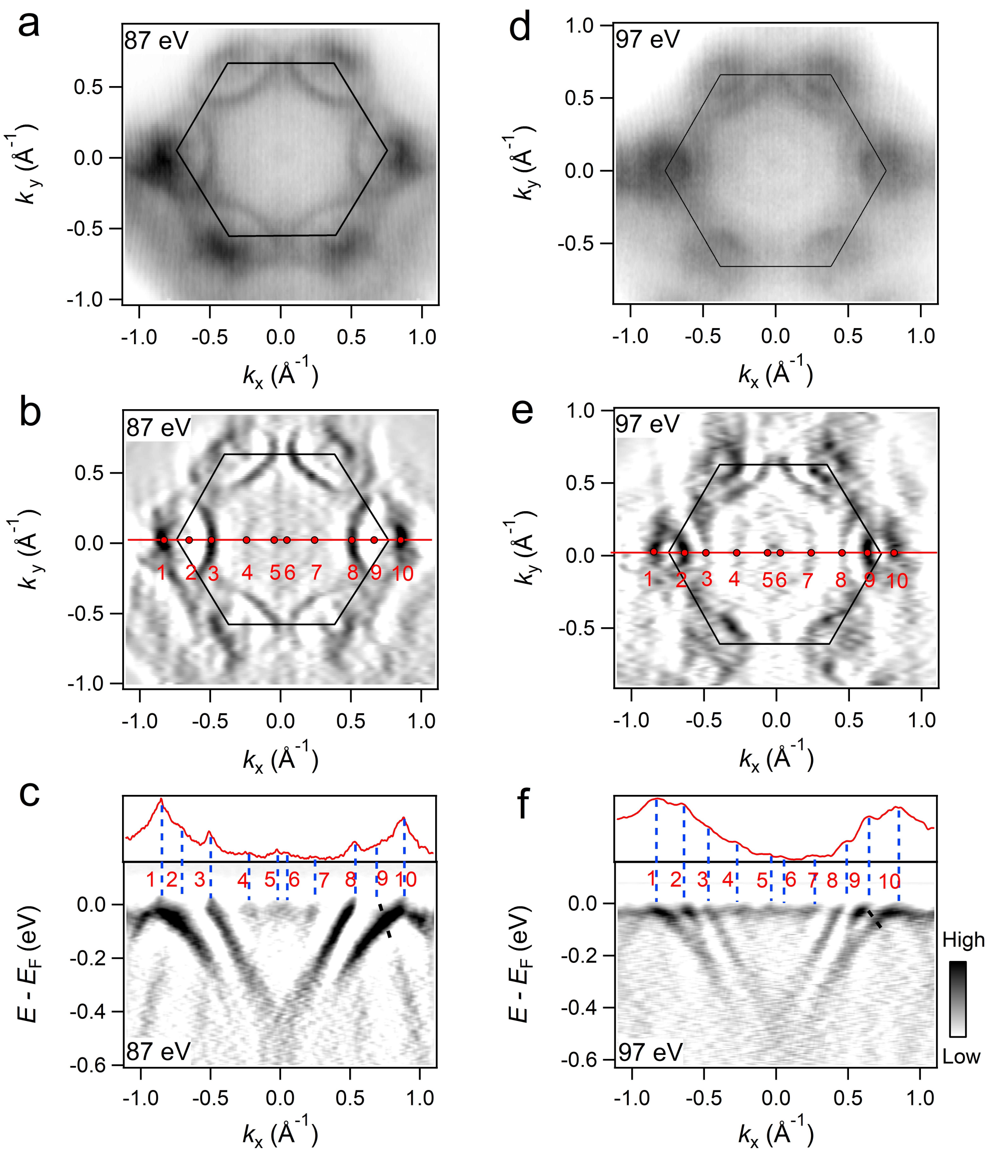

Supplementary Figure 2. (a) Fermi surfaces of YMn_6Sn_6 measured with $h\nu$ = 87 eV. The BZ is marked with the black solid hexagon. (b) The corresponding second derivative plot of (a). (c) Energy-momentum dispersions of YMn_6Sn_6 along the high-symmetry direction, marked as the solid red line in (b). Momentum distribution curve at Fermi energy is overlaid, with a dashed blue line indicating peaks at k_F. (d-f) Same as (a-c), but taken with 97-eV photons.

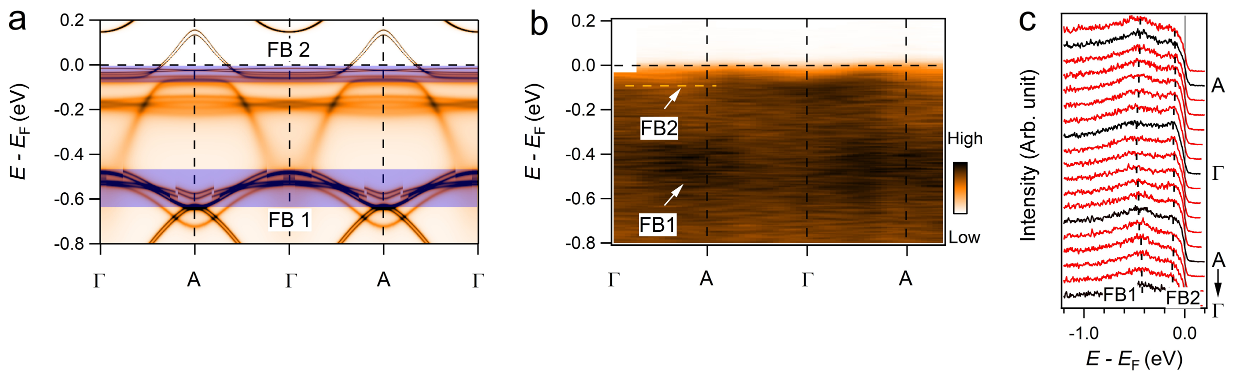

Supplementary Figure 3. (a) The DFT+DMFT calculation in the FM state with SOC along k_z. Flat bands, FB1 and FB2, are indicated by the color-shaded regions. (b) Intensity plot along the Γ–*A* direction in the in-plane-2nd BZ. The two flat bands are indicated by the white arrows. The dashed lines are served as guides for eyes. (c) EDCs plot of (b). The FB1 and FB2 are indicated by the black dashed lines.

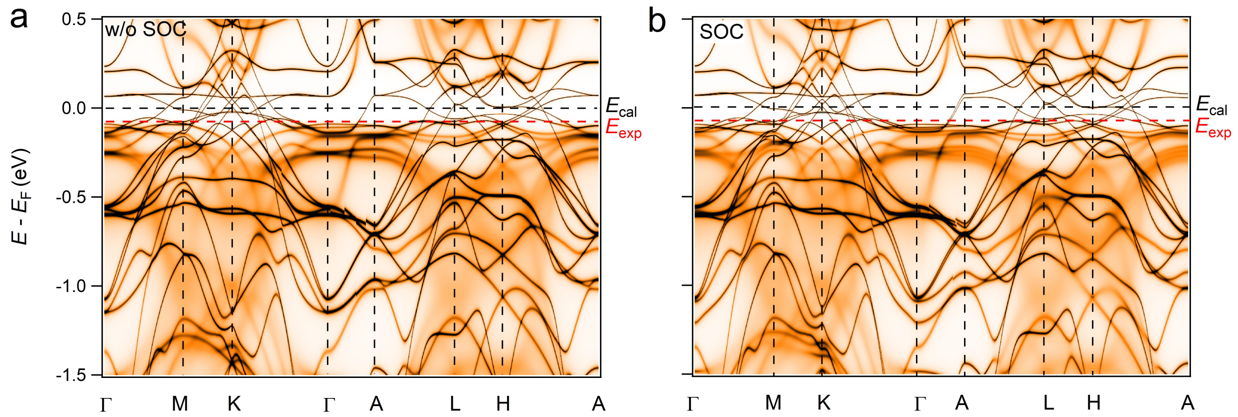

Supplementary Figure 4. The DFT+DMFT calculation of YMn_6Sn_6 in the FM state and (a) without SOC and (b) with SOC along the high-symmetry directions. The dashed red line indicates the experimental Fermi level.

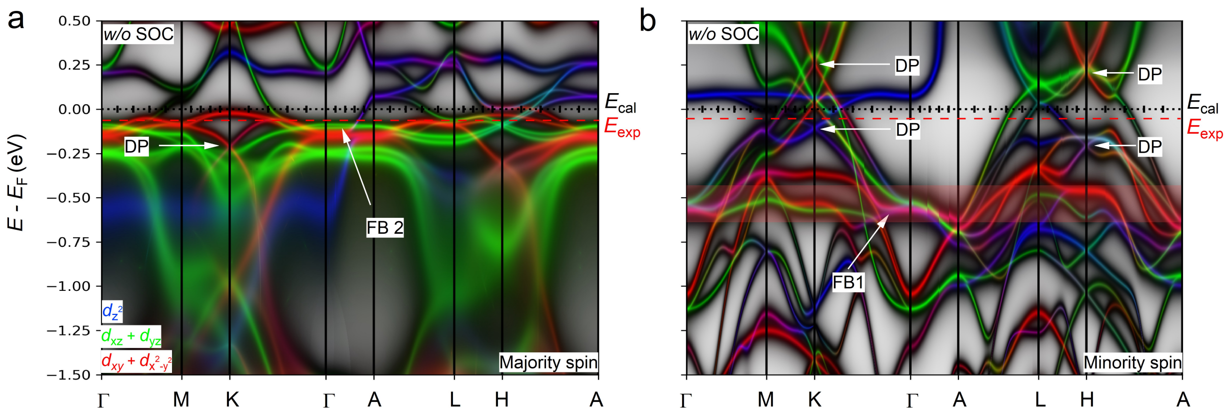

Supplementary Figure 5. The DFT+DMFT calculation with (a) spin-majority and (b) spin-minority states along the high-symmetry directions without SOC and with 3*d* orbitals projections. The red, green and blue lines indicate the orbitals components of $d_{xy}/d_{x^2-y^2}$, d_{xz}/d_{yz} and d_{z^2}, respectively. The magenta, cyan and light yellow are color mixings of red, green and blue, respectively.

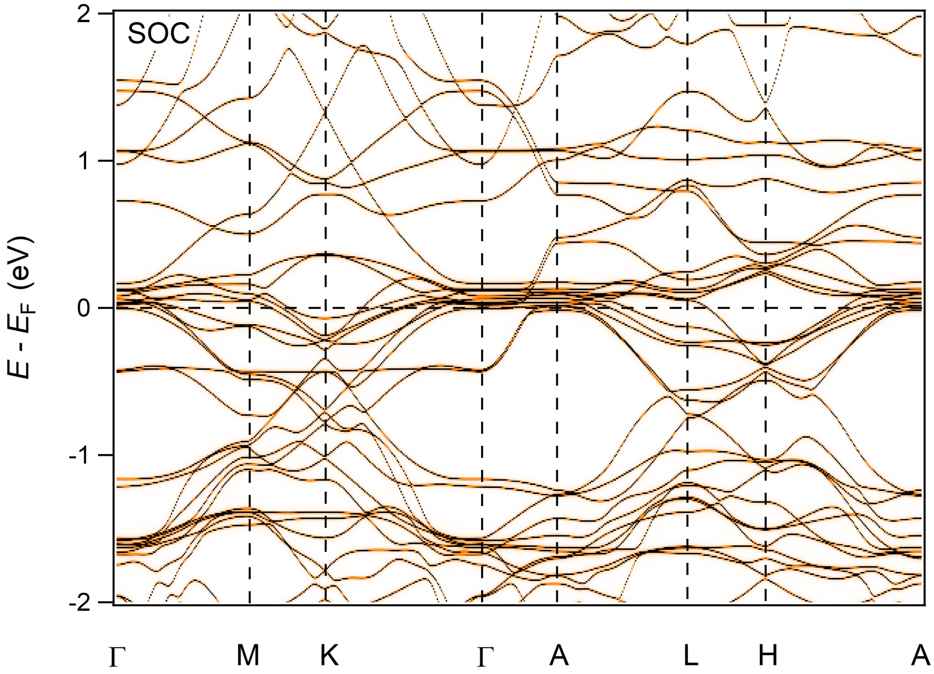

Supplementary Figure 6. The DFT calculation of YMn_6Sn_6 with SOC along the high-symmetry directions.

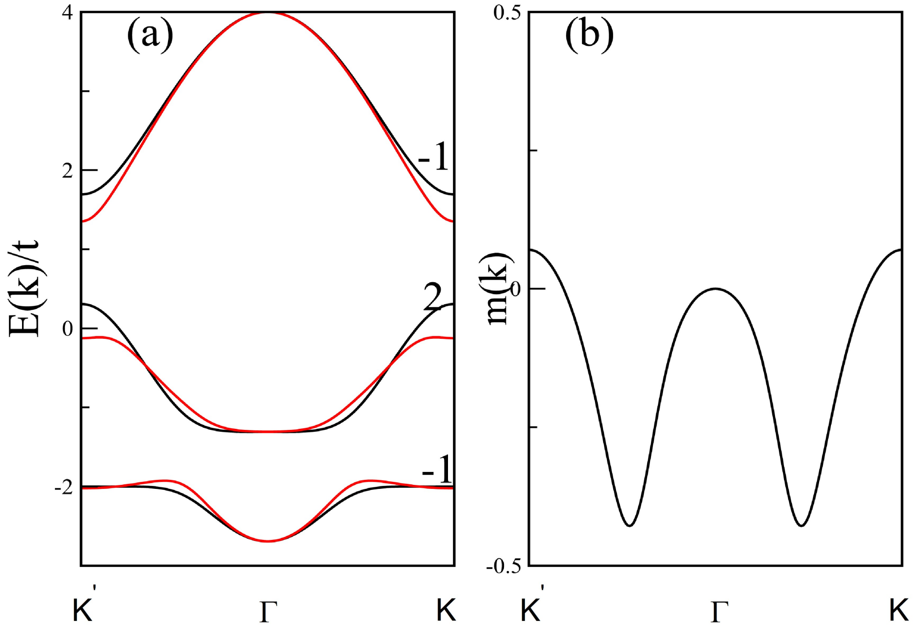

Supplementary Figure 7. (a)Band dispersion along $K' - \Gamma - K$ with $\lambda = 0.2t$. Chern numbers of each band are also shown. Red lines are bands coupling to $m(k)$. (b) $m(k)$ (unit of $\frac{e}{2\hbar}$) of the flat band in (a).

Supplementary Note 1. Sample growth and characterizations. Single crystals of YMn_6Sn_6 were grown by using Sn flux. Y lumps (purity 99.99 %), Mn granules (purity 99.9 %) and Sn grains (purity 99.99 %) with a molar ratio of Y : Mn : Sn = 1 : 6 : 30 put into an alumina crucible and sealed in a quartz ampoule under partial argon atmosphere. The sealed quartz ampoule was heated up to 1273 K and held for 24 hours. Then it was cooled down slowly to 873 K at a rate of 5 K/hour. Finally, the ampoule was taken out from the furnace and decanted with a centrifuge to separate YMn_6Sn_6 crystals from excess Sn flux. Magnetization and electrical transport measurements were carried out by using Quantum Design PPMS-14 T.

Supplementary Note 2. Angle-resolved photoemission spectroscopy experiments. ARPES measurement were performed at the Dreamline and 03U beamline of the Shanghai Synchrotron Radiation Facility (SSRF), and 1-squared ARPES end-station of BESSY. The combined energy resolutions were set to around 20 meV at low photon energies and 28 meV at high photon energies. The angular resolution is 0.2°. Samples were cleaved *in situ*, yielding a flat mirrorlike (001) surface. During the measurements, the temperature was kept at 25 K and the pressure was maintained less than 5×10^{-11} Torr.

We conducted core-level spectroscopy with the photon energy of 200 eV. In supplementary Fig. 1, we present the core-level photoemission intensity plot of YMn_6Sn_6 where the characteristic peaks of Y-3*d*, Sn-4*s*/4*d*, and Mn-3*s*/3*p* orbitals are clearly observed, with no extra peaks observed, suggesting the high quality of the crystal and the bulk origin with no surface contribution.

We measured the intensity plots and corresponding second derivative plots of YMn_6Sn_6 with 87-eV and 97-eV photons, as shown in supplementary Fig. 2. The high symmetry cuts with ten crossing points are observed between K–Γ–K line, as indicated with blue dotted lines along the solid red line of supplementary Fig. 2b,e. The Dirac cones locate on the marked between the ”1” and ”2” or ”9” and ”10”. One branch of the Dirac-like bands could be observed and another one is faint due to the matrix element effect associated with the chirality of the Dirac fermions similar to CoSn and FeSn [1, 2].

Supplementary Fig. 3 shows the intensity plots along Γ-A in the 2nd Brillouin zone (BZ). The FB2 is missed in the first BZ due to the matrix element effect, but clearly presented in the second BZ. The FB1 locates at ~ 0.4 eV below E_F with a narrow bandwidth (< 150 meV). The FB2 locates at ~ 0.06 eV below E_F showing nealy no dispersion along k_z.

Supplementary Note 3. Calculations. The electronic structures of YMn_6Sn_6 were computed by using density functional theory combined with dynamical mean field theory (DFT+DMFT) [3]. The density functional theory part is based on the full-potential linear augmented plane wave method implemented in Wien2K [4]. The Perdew-Burke-Ernzerhof generalized gradient approximation [5] is used for the ex-

change correlation functional. DFT+DMFT was implemented on top of Wien2K and was described in details in Ref. 6. In the DFT+DMFT calculations, the electronic charge was computed self-consistently on DFT+DMFT density matrix. The quantum impurity problem was solved by the continuous time quantum Monte Carlo (CTQMC) method [7, 8] with a Hubbard U=4.0 eV and Hund's rule coupling J=0.7 eV. The experimental crystal structure [9] (space group P6/mmm, No. 191) of YMn_6Sn_6 with lattice constants a=b=5.512 Å and c=8.984 Å was used in the calculations.

Supplementary Note 4. Orbital magnetism. The orbital magnetism of a wave packet is related to its angular momentum [10, 11],

$$\mathbf{m_n}(\mathbf{k}) = -i\frac{e}{2\hbar}\langle\nabla_{\mathbf{k}}u_n| \times [H(\mathbf{k}) - \epsilon_n(\mathbf{k})]|\nabla_{\mathbf{k}}u_n\rangle \tag{1}$$

$$= -i\frac{e}{2\hbar}(\langle\frac{\partial u_n}{\partial k_x}|H(\mathbf{k}) - \epsilon_n(\mathbf{k})|\frac{\partial u_n}{\partial k_y}\rangle - c.c.)\mathbf{e_z} \tag{2}$$

$$= -i\frac{e}{2\hbar}\sum_{m\neq n}[\frac{\langle u_n|\frac{\partial H(\mathbf{k})}{\partial k_x}|u_m\rangle\langle u_m|\frac{\partial H(\mathbf{k})}{\partial k_y}|u_n\rangle}{\epsilon_m(\mathbf{k}) - \epsilon_n(\mathbf{k})} - c.c.]\mathbf{e_z} \tag{3}$$

where n, m are band indices. The derivation of Eq.3 can be found in Ref [12], which is similar to the equations of Berry curvature $\mathbf{\Omega}_n(\mathbf{k})$ [12, 13].

$$\mathbf{\Omega}_n(\mathbf{k}) = i\langle\nabla_{\mathbf{k}}u_n| \times |\nabla_{\mathbf{k}}u_n\rangle \tag{4}$$

$$= i(\langle\frac{\partial u_n}{\partial k_x}|\frac{\partial u_n}{\partial k_y}\rangle - \langle\frac{\partial u_n}{\partial k_y}|\frac{\partial u_n}{\partial k_x}\rangle) \tag{5}$$

$$= i\sum_{m\neq n}[\frac{\langle u_n|\frac{\partial H(\mathbf{k})}{\partial k_x}|u_m\rangle\langle u_m|\frac{\partial H(\mathbf{k})}{\partial k_y}|u_n\rangle}{(\epsilon_m(\mathbf{k}) - \epsilon_n(\mathbf{k}))^2} - c.c.] \tag{6}$$

The only change of $\mathbf{\Omega}_n(\mathbf{k})$ and $\mathbf{m_n}(\mathbf{k})$ is the extra factor of $H(\mathbf{k}) - \epsilon_n(\mathbf{k})$ in the numerator cancels a $\epsilon_m(\mathbf{k}) - \epsilon_n(\mathbf{k})$ in the denominator.

Supplementary Note 5. Kagome lattice with Kane-Mele SOC and Ferromagnetism. We consider a unit cell of the kagome lattice spaned by the primitive lattice $2\mathbf{a_1} = (2, 0)$,$2\mathbf{a_2} = (1, \sqrt{3})$ and define $\mathbf{a_3} = \mathbf{a_2} - \mathbf{a_1}$. In the ferromagnetic kagome lattice with SOC strength λ, the spinless Hamiltonian in k-space is

$$H(k) = 2t\begin{bmatrix} 0 & \cos\mathbf{k}\cdot\mathbf{a_1} & \cos\mathbf{k}\cdot\mathbf{a_2} \\ \cos\mathbf{k}\cdot\mathbf{a_1} & 0 & \cos\mathbf{k}\cdot\mathbf{a_3} \\ \cos\mathbf{k}\cdot\mathbf{a_2} & \cos\mathbf{k}\cdot\mathbf{a_3} & 0 \end{bmatrix} + 2\lambda i\begin{bmatrix} 0 & \cos\mathbf{k}\cdot(\mathbf{a_2}+\mathbf{a_3}) & -\cos\mathbf{k}\cdot(\mathbf{a_3}-\mathbf{a_1}) \\ -\cos\mathbf{k}\cdot(\mathbf{a_2}+\mathbf{a_3}) & 0 & \cos\mathbf{k}\cdot(\mathbf{a_1}+\mathbf{a_2}) \\ \cos\mathbf{k}\cdot(\mathbf{a_3}-\mathbf{a_1}) & -\cos\mathbf{k}\cdot(\mathbf{a_1}+\mathbf{a_2}) & 0 \end{bmatrix} \tag{7}$$

We choose $\lambda = 0.2t$. The dispersion along $K' - \Gamma - K$ is shown in supplementary Fig. 7(a). Orbital magnetism $\mathbf{m_n}(\mathbf{k})$ of the flat band is shown in supplementary Fig. 7(b).

Supplementary references

---

[1] Kang, M. *et al.* Dirac fermions and flat bands in the ideal kagome metal FeSn. *Nat. Mater.* **19**, 163–169 (2020).

[2] Kang, M. *et al.* Topological flat bands in frustrated kagome lattice CoSn. *Nat. Commun.* **11**, 4004 (2020).

[3] Kotliar, G. *et al.* Electronic structure calculations with dynamical mean-field theory. *Rev. Mod. Phys.* **78**, 865 (2006).

[4] Blaha, P., Schwarz, K., Madsen, G., Kvasnicka, D. & Luitz, J. WIEN2K, An Augmented Plane Wave+Local Orbitals Program for Calculating Crystal Properties. *Karlheinz Schwarz, Techn. Universität Wien, Austria* (2001).

[5] Perdew, J. P., Burke, K. & Ernzerhof, M. Generalized gradient approximation made simple. *Phys. Rev. Lett.* **77**, 3865–3868 (1996).

[6] Haule, K., Yee, C.-H. & Kim, K. Dynamical mean-field theory within the full-potential methods: Electronic structure of $CeIrIn_5$, $CeCoIn_5$, and $CeRhIn_5$. *Phys. Rev. B* **81**, 195107 (2010).

[7] Haule, K. Quantum monte carlo impurity solver for cluster dynamical mean-field theory and electronic structure calculations with adjustable cluster base. *Phys. Rev. B* **75**, 155113 (2007).

[8] Werner, P., Comanac, A., de Medici, L., Troyer, M. & Millis, A. J. Continuous-time solver for quantum impurity models. *Phys. Rev. Lett.* **97**, 076405 (2006).

[9] Malaman, B., Venturini, G. & Roques, B. *Mater. Res. Bull.* **23**, 1629–1633 (1988).

[10] D. Xiao, Q. N., M. C. Chang. *Rev. Mod. Phys.* **82**, 1959 (2010).

[11] D. Xiao, W. Y. & Niu, Q. *Phys. Rev. Lett.* **99**, 236809 (2007).

[12] Chang, M. C. & Niu, Q. *Phys. Rev. B* **53**, 7010 (1996).

[13] T. Thonhauser, I. J. *Mod. Phys. B* **25**, 1429 (2011).
